# Implications of Obstructive Sleep-related Breathing Disorder in Dentistry: Focus on Snoring and Obstructive Sleep Apnea

**DOI:** 10.26502/droh.0051

**Published:** 2022-10-05

**Authors:** Yeon-Hee Lee

**Affiliations:** Department of Orofacial Pain and Oral Medicine, Kyung Hee University Dental Hospital, Kyung Hee Medical center, Kyung Hee University, Seoul, Korea

**Keywords:** Sleep-related breathing disorder, Snoring, Obstructive sleep apnea, Temporomandibular disorder, Orofacial pain, Dentistry

## Abstract

Obstructive sleep-related breathing disorder (SRBD) is an umbrella term that encompasses various types of upper airway dysfunctions during sleep characterized by increased respiratory effort secondary to snoring and/or increased upper airway resistance and pharyngeal collapse. Obstructive sleep apnea (OSA) is a representative SRBD that involves a significant decrease in or cessation of airflow despite the presence of respiratory effort. While snoring is considered a normal condition, it can cause serious noise disturbance to sleep partners and is considered a predictor of OSA. Snoring and OSA are highly correlated with obesity. SRBDs can lead to cardiovascular disease, hypertension, decreased quality of life, decreased work efficiency, daytime sleepiness, decreased neurocognitive activity, and psychological impairments. In dentistry, research on sleep problems has focused on temporomandibular disorder (TMD)/orofacial pain. The relationship between OSA and TMD/orofacial pain has been reported, but it is not clear whether it is a simple correlation or a causal relationship. Therefore, we aimed to review the causes of SRBDs including snoring and OSA and to review and infer the relationship between these SRBDs and TMD/orofacial pain. The effects of snoring and OSA extend beyond sleep disturbances and are worthy of future research, especially with regard to TMD.

## Introduction

Sleep and pain have a bidirectional relationship. In other words, disturbances in sleep exacerbate pain or lead to persistent pain, and the presence of pain interferes with sleep [1]. During sleep, upper airway patency decreases, and upper airway resistance on ventilation increases. Obstructive sleep-related breathing disorder (SRBD) is a broad term encompassing various upper airway dysfunctions during sleep characterized by increased respiratory effort secondary to snoring and/or greater upper airway resistance and pharyngeal collapse [2]. SRBD is a group of disorders including upper airway resistance syndrome, central sleep apnea, and obstructive sleep apnea (OSA). SRBD can lead to cardiovascular disease, hypertension, decreased quality of life, decreased work efficiency, daytime sleepiness, deteriorated neurocognitive activity, increased systemic inflammation, and psychological impairment [3]. OSA is a representative SRBD with crucial implications beyond interference with sleep partners. OSA is a sleep disorder in which airflow is significantly reduced or stopped in the presence of respiratory effort [4,5]. Patients with OSA experience collapse of the upper airway during sleep, which is a hallmark sign. OSA is an independent risk factor for cardiovascular, neurological, and psychological morbidities. Although snoring is considered a physiologically normal condition with noisy sounds, such as a growling or gasping sound, it can cause serious noise disturbance for sleep partners and can be a predictor of OSA [6,7]. In general, snoring and OSA are highly correlated with obesity and anatomical narrowing of the upper airway. In patients with OSA, hypoxemia and sleep segmentation during sleep can activate the sympathetic nervous system and increase oxidative stress. Inflammatory responses are assumed to be the main mechanisms responsible for the diseases mentioned above. In dentistry, the relationship between OSA, systemic inflammation, and temporomandibular disorder (TMD)/orofacial pain has been reported [8]. There is still a lack of evidence on whether a simple correlation, causal relationship, or comorbidity exists between OSA and TMD/orofacial pain. Therefore, this narrative review aimed to review the etiology of obstructive SRBD including snoring and OSA, to explain the pathophysiology of obstructive sleep disorder respiration, and to review and infer the relationship between OSA and TMD/orofacial pain. This review highlights that the effect of SRBDs, such as snoring and OSA, expands beyond sleep-related problems and is worth examining in future research on TMD.

## Materials and Methods

A narrative review was performed based on a search of the PubMed and Google Scholar databases for articles on the etiology, pathophysiology, and role of obstructive SRBDs, including snoring and OSA, in dentistry. The following keywords were used in the search to find related articles: “sleep-related breathing disorder,” “snoring,” “sleep apnea,” “obstructive sleep apnea,” “obstructive sleep apnea syndrome,” “sleep,” “temporomandibular joint (TMJ),” “orofacial,” “orofacial pain,” and “TMD”. Papers published in English over the last 30 years (between January 1992 and August 2022) were searched. A total of 2618 articles were retrieved from the PubMed and Google Scholar databases over the 30-year period from January 1992 to August 2022. After application of the inclusion and exclusion criteria and analyzing the abstracts and full texts of some articles, 82 articles were finally selected. In this review, data on OSA were also taken from articles on OSA syndrome, SRBDs, and sleep apnea, if relevant. In other words, for the purpose of this review, “OSA” included all types of OSA except for central sleep apnea and Cheyne-Stokes breathing. The author repeatedly reviewed the papers over a 3-week period to verify the content and study design and determine whether the papers were suitable for inclusion in this study.

## Results

### Diagnosis and pathophysiology of snoring

Snoring disorder can occur in anyone and at any age. Snoring can also be a predictor of chronic problems and severe SRBDs. Snoring refers to the sound produced by vibrations of respiratory structures of the upper airway tract during sleep [9]; snoring is caused by vibrations of the oropharynx structure when air passes through relaxed tissues of the nose and throat. Snoring without daytime sleepiness, fatigue, or OSA is simple snoring [10]. According to the International Classification of Sleep Disorders, 3rd edition (ICSD-3), snoring is classified as an SRBD [11].

Snoring can impair bed partners’ sleep as an acoustic disturbance and a potential source of noise pollution. According to previous reports, the prevalence rate of snoring is between 3.8% and 40.3%, and it increases with age [12]. Snoring increased with age and peaked at ages 50–59 in both men and women. In women, menopause was associated with the occurrence of snoring, and women aged 59 and older experienced a less dramatic decrease in snoring than men [13].

The etiologies of snoring are diverse and complex, including anatomical problems of the mouth (thick soft palate and narrow airways) and nose (septum dislocation and chronic nasal congestion), sinusitis, allergies, poor sleep posture, weight gain, overweight, obesity, alcohol consumption, and hormonal changes [14]. Typical anatomical features of obstructive sleep apnea (OSA) in the orofacial area are: (1) an abnormally enlarged tongue; (2) the long soft palate and/or tonsillar enlargement; (3) an extended uvula; (4) high palatal vault; (5) posteriorly positioned mandible (retrognathia) or small-sized mandible; (6) loss of normal occlusion; (7) adenoid hypertrophy; (8) reduced pharyngeal upper airway space; (9) the increased distance between the mandible and the hyoid bone; and (10) increased neck fat deposition surrounding the upper airway (Figure 2) [15,16]. According to Ohayon et al., regular snoring was significantly associated with age >25 years, male sex, obesity, smoking, daytime sleepiness, sleep fragmentation, and high caffeine intake [12]. Snoring can be detected and/or diagnosed using polysomnography, reports from sleep partners, or snoring recording [17].

Anatomical upper airway obstruction may indicate snoring with OSA [18]. In a Hungarian population survey, 37% of male and 21% of female participants with loud snoring had sleep apnea [19]. Snoring and OSA are risk factors for cardiovascular disease, and noise pollution from snoring in excess of 53 dB can cause adverse cardiovascular events in both snorers and sleep partners [20]. In particular, accumulated nighttime exposure to snoring may contribute to the onset and/or progression of cardiovascular disease [21]. Cardiovascular stress increases sympathetic activation, which can lead to spikes in the heart rate and persistently elevated blood pressure during sleep [22]. Furthermore, when SRBDs disrupt the regulation of inflammation by the sympathetic nervous system and neurotransmitters, systemic inflammatory reactions may become uncontrolled or persistent [23]. This can inevitably worsen pain conditions. Nevertheless, objective investigations of the association between snoring severity and OSA are lacking.

### Diagnosis of OSA

**Figure 1:**
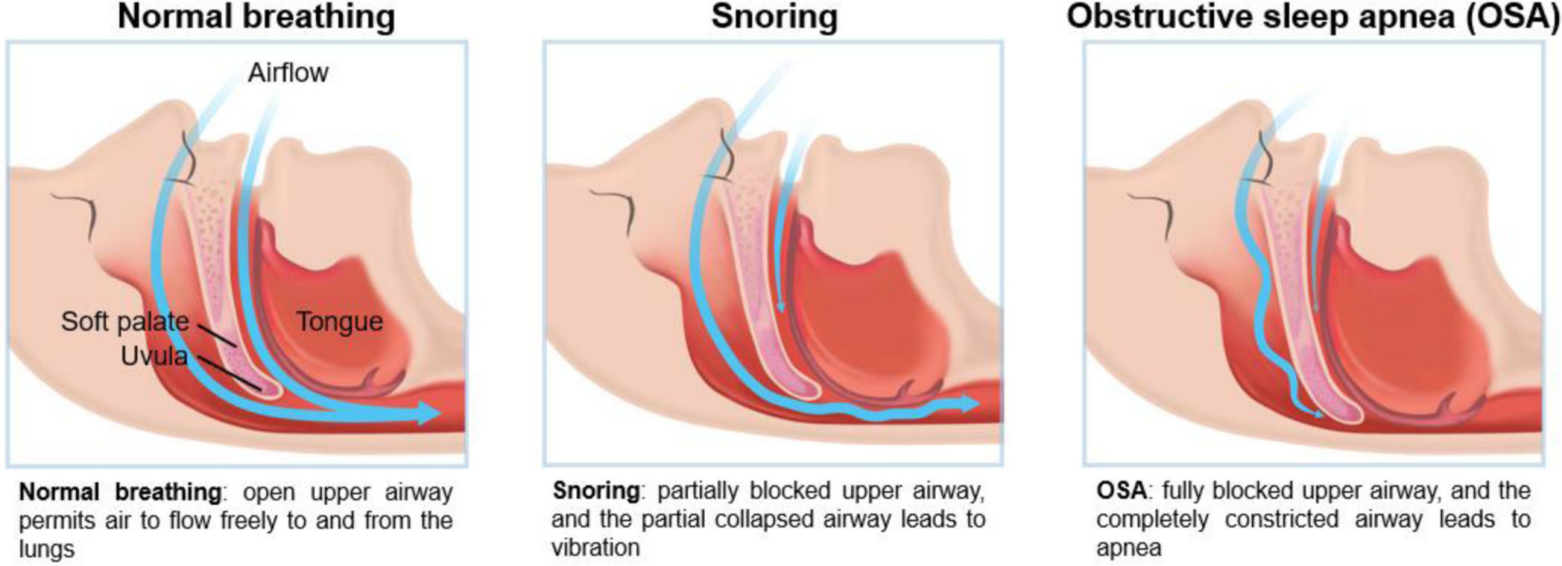
Schematic of snoring and obstructive sleep apnea compared to normal breathing

**Figure 2:**
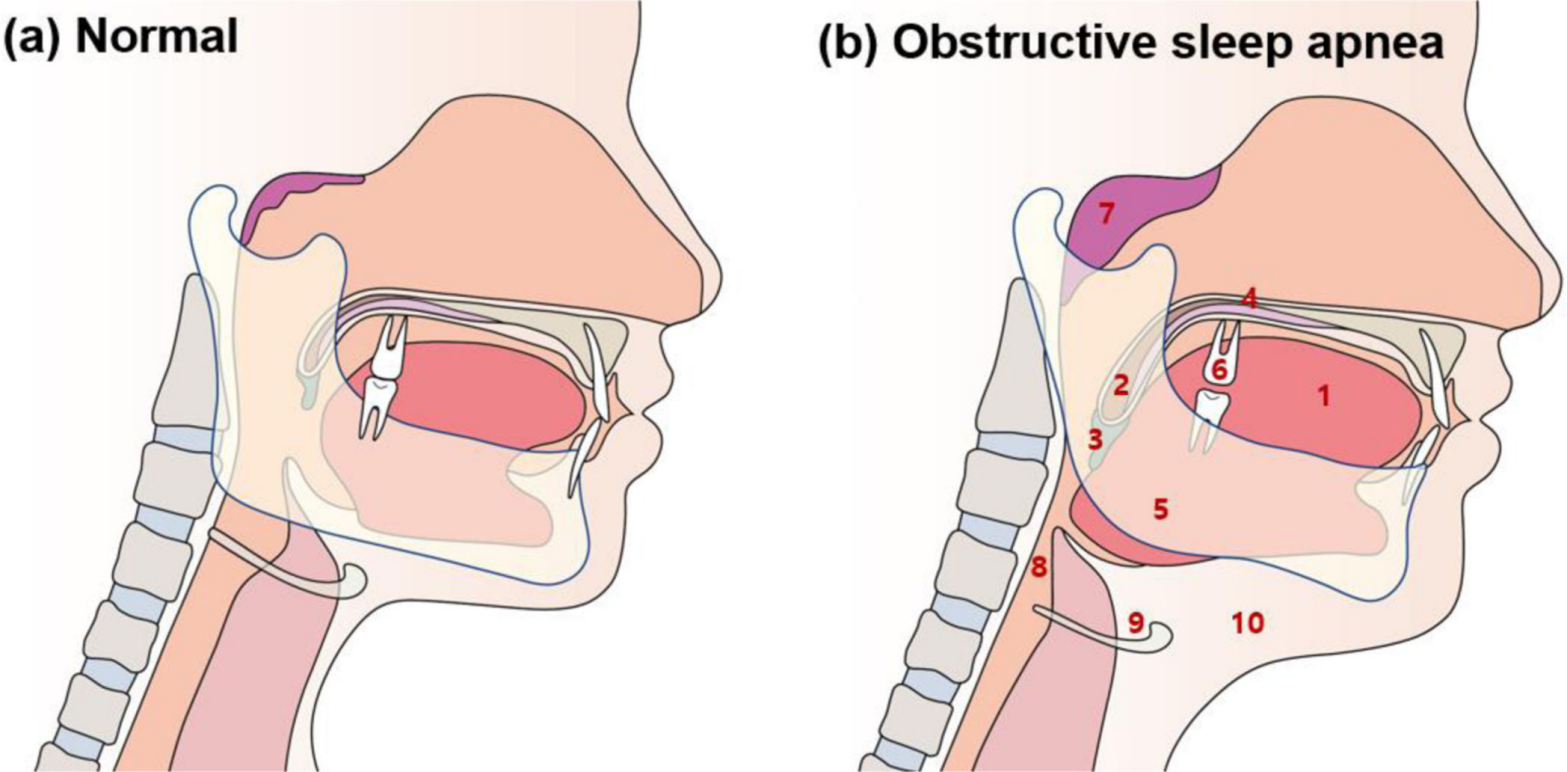
Typical anatomical features of obstructive sleep apnea in the orofacial area

OSA is characterized by recurrent episodes with cessation of airflow (apnea) or shallow breathing (hypopnea) during sleep despite the presence of respiratory efforts (Figure 1). When the duration of a ≥90% airflow drop in the sensor signal is ≥10 seconds, it counts as an occurrence of an apnea event. The technical definition of hypopnea is shallow breathing lasting more than 10 seconds in which an airflow is reduced by ≥30% [24]. Consequently, hypoxemia, arousal, sleep segmentation, and sympathetic hyperactivity occur repeatedly during sleep [25]. Respiratory effort related arousal (RERA) can occur in patients with OSA. RERA is scored when the inspiratory nasal pressure is flattened for more than 10 seconds leading to arousal from sleep. Arousals in RERA events occur due to respiratory effort, but does not meet the criteria for apnea or hypopnea [26]. The severity of OSA is determined by three representative objective indices. For SRBD and OSA diagnosis, (1) apnea hypopnea index (AHI) or (2) respiratory disturbance index (RDI) can be used if polysomnography is performed, and (3) respiratory event index (REI) can be used if out-of-center sleep testing was accompanied [27].

Each OSA index is derived by the following formula:

- AHI = number of apneas+hypopneas/total sleep time
- RDI = number of apneas+hypopneas +RERAs/total sleep time
- REI = number of apneas+hypopneas/monitoring time

When microarousals or arousals occur in RERA, respiratory effort in OSA patients can be resolved. OSA is a common disease with a prevalence of 3–5%, with asymptomatic patients accounting for up to 26% of the patient population [28]. In the general population, its prevalence is approximately 4% in men and 2% in women [29]. OSA causes excessive daytime sleepiness, fatigue, drowsiness, poor concentration, cognition, and memory loss in daily life. Various factors are associated with OSA: (1) structural factors, such as adenotonsillar hypertrophy, craniofacial abnormality, and obesity; (2) neuromotor factors, such as cerebral palsy and genetic diseases; and (3) other factors, such as hormonal changes and aging. Obesity is a well-known risk factor for OSA [30]. In patients with OSA, apnea and hypopnea during sleep cause hypoxemia, hypercapnia, and frequent arousal, resulting in poor sleep quality at night [31]. Furthermore, OSA has been reported to affect the incidence of depression, psychological disorders, diabetes, coronary artery disease, stroke, pulmonary hypertension, and cardiovascular mortality [32,33].

OSA, the most common subtype of SRBDs, accounts for 95% of all apnea cases [34] but is poorly diagnosed. OSA is currently diagnosed according to the ICSD-3 diagnostic criteria using polysomnography [11]. The detailed OSA criteria of ICSD-3 define OSA as follows: (1) ≥5 respiratory events per hour on a sleep test and accompanying clinical symptoms or comorbidities such as cardiovascular disease and (2) >15 respiratory events per hour on a sleep test [11]. OSA is generally defined as AHI or REI ≥5 [27]. AHI is the sum of the number of apneas and hypopneas per hour during sleep. According to the American Academy of Sleep Medicine, OSA is categorized as normal (<5 events/h), mild OSA (5–15 events/h), moderate (15–30 events/h), and severe (>30 events/h) [35]. Vulnerability to excessive daytime sleepiness varies with the severity of AHI [36]. Polysomnography is the gold standard for the diagnosis of OSA. Level I polysomnography, performed in the presence of a test facilitator, is the most definitive diagnostic method for OSA [37].

The most common symptom in clinical examination of the patients is snoring, which may suggest OSA [38]. Simple snoring may be due to mild obstruction of the upper airway, which is a possible risk factor for the development and progression of OSA. In addition, sleep apnea, in which breathing repeatedly ceases and then resumes, is observed by sleep partners. About a quarter of the patients with OSA complain of daytime sleepiness and various signs or symptoms such as awakening during sleep due to choking or gasping, nocturia, headache after waking up, poor concentration, sensitivity, depression, insomnia, and impotence [39].

On physical examination of patients with OSA, signs such as an increase in waist or neck circumference accompanying obesity or overweight can be observed [40]. A deviated nasal septum, hypertrophy of the turbinate, and a vertically low position of the hyoid bone are also related to the severity of OSA [41]. In addition, the size of the tonsils or upper airway restriction can be confirmed using the Mallampati score or Friedman stage evaluation method [42,43]. The Berlin Questionnaire and STOP-Bang questionnaire are some simple test tools for screening high-risk patients with OSA [44]. The Epworth Sleepiness Scale is commonly used to evaluate daytime sleepiness in patients with OSA [45]. These questionnaires are used as simple tools for screening high-risk groups rather than for diagnosing OSA.

### Pathophysiology of OSA

OSA is a heterogeneous syndrome that results from various predispositions, clinical characteristics, respiratory events, and pathophysiological mechanisms. In OSA, recurrent episodes of apnea and hypopnea result in a decrease in oxyhemoglobin saturation and sleep fragmentation and a decrease in the amount of slow waves and rapid eye movement sleep [46]. Although anatomical collapsibility of the upper airway is a very important etiologic factor in apnea and hypopnea events, it is also a consequence of non-anatomical causes and thus cannot be attributed to a single anatomical cause [47]. Phenotypes of OSA are classified according to the major mechanisms of disease development and degrees of anatomical collapsibility of the upper airways, loop gain, and arousal threshold [48].

Regarding anatomical collapse of the upper airway as a mechanism of OSA, the main aspects include narrowing of the upper airway due to anatomical structural problems of the upper airway, collapsing of the oropharynx, and deteriorating pharyngeal patency due to obesity with fat accumulation in soft tissues and the tongue [49]. In addition, the increase in central adipose tissue due to abdominal obesity can increase the collapsibility of the pharynx by reducing lung volume [50]. Collapse of the upper airway during sleep and its collapsibility can be evaluated using the critical closing pressure (Pcrit) method. However, the range of Pcrit in patients with OSA varies, and 20% of patients show values similar to those of the normal individuals, suggesting that OSA is not just a problem of upper airway collapsibility [51]. Commonly used traditional OSA treatments such as continuous positive airway pressure, mandibular advancement device, upper airway surgery, weight loss, and positional therapy can help resolve the issue of anatomical collapsibility [52,53].

However, recent studies have suggested that non-anatomical mechanisms may play an important role in the pathophysiology of OSA. Gray et al. claimed that at least one non-anatomical mechanism contributes to the development of OSA in approximately 70% of patients with OSA [54]. The key factor to be considered in the occurrence of OSA due to non-anatomical mechanisms is the function of muscles of the pharynx and the neuromodulatory action on these muscles [55]. Muscles of the pharynx are important in the development and progression of OSA as they play a key role in keeping the upper airway open.

Another non-anatomical mechanism of OSA is related to lowered arousal threshold. When the increase in negative pressure in the thoracic cavity reaches a certain threshold, cortical awakening occurs during a sleep apnea event [56]. Arousal threshold refers to the degree of breathing effort during arousal. OSA elicits cortical arousal during sleep [57]. Conversely, a lowered arousal threshold is commonly related to OSA occurrence or frequent brief awakenings [58]. Sleep and arousal events are repeated periodically during sleep in patients with OSA, and these repetitions make breathing unstable and interfere with deep sleep, exacerbating OSA [59].

Finally, the non-anatomical mechanism is related to loop gain, which is a control mechanism of human respiration. Loop gain has been used to quantify the internal amplification of a system [60]. In other words, the dimensionless value of the propensity of a system to be controlled by a feedback loop to develop unstable behavior is called the loop gain. The concept of loop gain can be applied to the respiratory system. In the respiratory system of patients with high loop gain, quantified as the ratio of ventilatory response to total respiratory disturbance, ventilatory control is unstable, resulting in an excessive ventilatory response to small changes in CO_2_ [61,62]. Ventilatory overreaction can lead to hypocapnia, which can worsen sleep apnea by reducing the respiratory drive [63].

### Relationship between SRBD and TMD/orofacial pain

**Figure 3:**
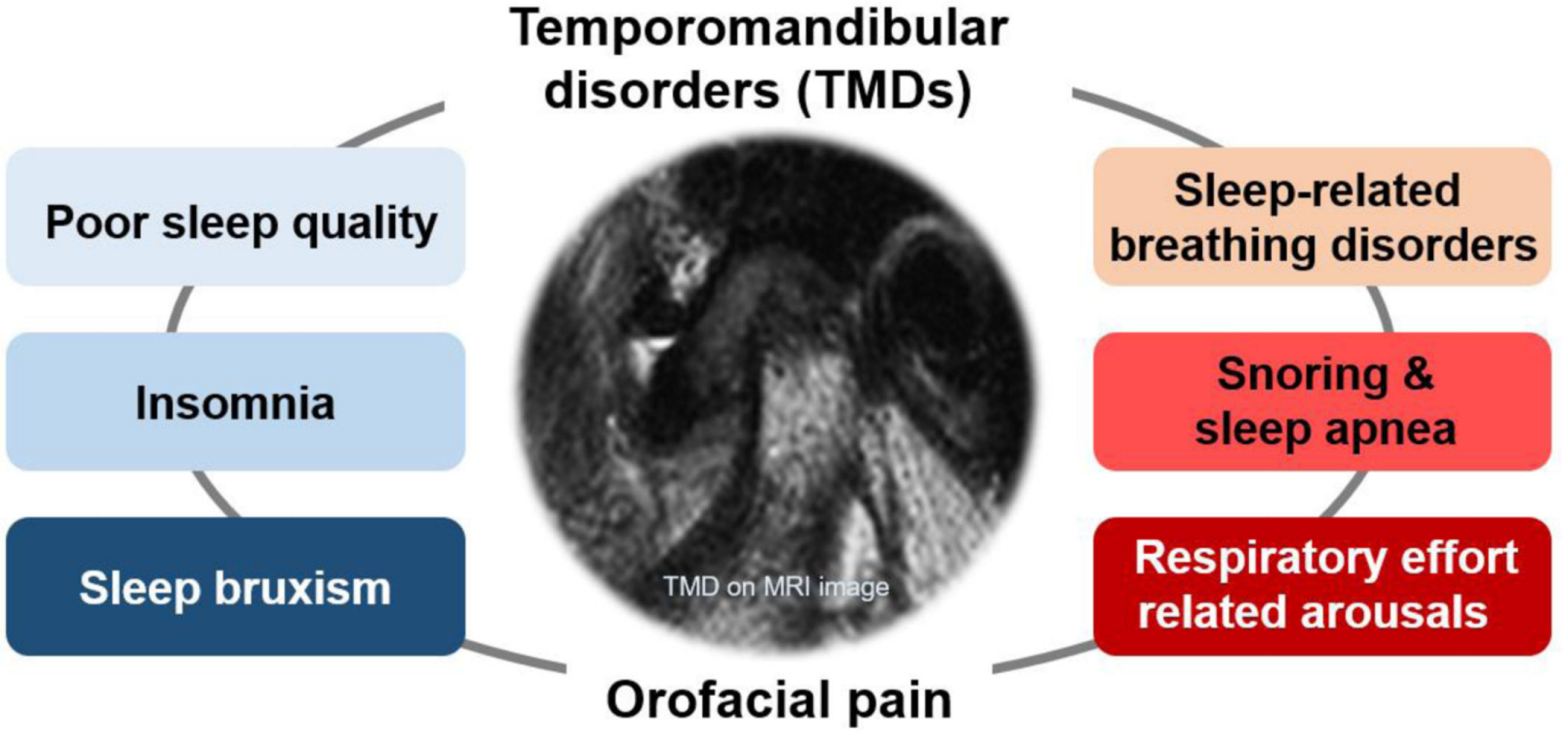
Temporomandibular disorder and sleep problems

TMD is an umbrella term characterized by clinical pain and dysfunction involving the TMJ, masticatory muscles, and their adjacent related structures [64]. TMD is very common in the general population, with a reported prevalence of up to 15% in adults [65]. Possible risk factors for TMD include parafunctional oral habits, macrotrauma, microtrauma, psychological problems, other bodily pain conditions, and sleep problems [64]. The etiopathophysiology of TMD is complex, and TMD is difficult to treat/manage and prone to becoming chronic. Approximately 50% of people with self-reported low sleep quality have comorbid chronic pain [66]. According to the diagnostic criteria for TMD, disc displacement, joint pain, myofascial pain, degenerative and inflammatory joint disease, and headaches attributed to TMD are the major and common subtypes of TMD [67]. Deterioration of sleep quality has been reported in 90% of patients with TMD [68]. At high co-occurrence rates, a close, bidirectional relationship between TMD pain or orofacial pain and sleep disorders can be inferred (Figure 3). In addition, orofacial pain, including TMD pain, and sleep deterioration may have significantly overlapping etiopathophysiologies or underlying mechanisms [68,69]. Although these diseases or conditions may occur independently in some patients, it should be considered that they may occur together in causal relationships or comorbidities in other patients, particularly in patients with chronic disease such as TMD. Among the patients with TMD pain, the number of poor sleepers was significantly higher in the TMD pain (76.8%) and myalgia groups (71.7%) than in the arthralgia group (54.8%) [70]. It has been found that myofascial pain in TMD is associated with elevated sleep fragmentation and increased frequency of RERA events [71]. The effect of TMD on sleep deterioration may differ, depending on the origin of TMD pain. However, further studies are needed to elucidate the relationship between the origin of TMD pain and sleep quality and their underlying mechanisms.

A significant relationship between OSA and TMD can be inferred from the high incidence of OSA in patients with TMD and vice versa. In the OPPERA (Orofacial Pain: Prospective Evaluation and Risk Assessment) cohort study on TMD and orofacial pain, loud snoring was a contributing factor to a high risk of OSA in patients with TMD [72]. However, few studies have examined the relationship between TMD and snoring as a subtype of SRBD, and there are only a few papers on OSA and TMD. In a population-based cohort of adults without TMD at baseline, baseline OSA signs/symptoms of the participants were associated with the incidence of first-onset TMD [73]. In an earlier study using polysomnography, out of 87 adults with mild or moderate OSA, 32 (36.8%) had TMD according to the research diagnostic criteria for TMD (RDC/TMD) [74]. Among 53 patients with myofascial TMD pain diagnosed using the RDC/TMD, 28% had OSA on polysomnography [74]. The effect of snoring on orofacial pain is not clear, with very few studies on this topic [75]. Only a few papers have been published on the relationship between SRBD and TMD. The relationship between these elements is still controversial, and further studies are needed.

The immediate effects of OSA on the body are persistent sympathetic excitation, oxyhemoglobin desaturation, blood pressure and heart rate fluctuations, cortical arousal, and sleep fragmentation [76]. The long-term effects of OSA may include various cardiovascular diseases, systemic hypertension, neurocognitive impairment, metabolic syndrome, morbidities, sociopsychological impairment, and chronic pain conditions [77,78]. The possible mechanisms by which SRBD may contribute to pain over time include pain amplification with decreasing functioning of the pain inhibitory systems and peripheral and central sensitizations [79]. A close relationship between sleep, pain, and central sensitization has been reported [80]. Sleep bruxism is a rhythmic masticatory muscle activity during sleep that is weakly correlated with certain parameters of OSA [81]. Sleep bruxism may ultimately increase myofascial pain in patients with TMD [82]. With regard to OSA and repeated facial movements, lack of adequate rest between muscle activities can lead to overload of related muscles and/or the TMJ. Moreover, SRBD-induced systemic sympathetic hyperactivity, increased inflammatory response, and secondary socio-psychological damage are highly likely outcomes. Further studies are required to investigate the mechanisms underlying the relationship between SRBD and TMD or the co-factors shared by two diseases such as psychological impairment and sleep bruxism.

### Study limitations

This study aimed to determine the relationship between SRBDs and TMD, with a particular focus on snoring and OSA. However, there are insufficient statistically relevant data to draw specific conclusions regarding this relationship, and there is still a lack of controlled clinical trials or multicenter studies and randomized controlled trials on this issue. Since there are few original articles on the relationship between SRBD or TMD, it is difficult to determine the exact negative effects and underlying mechanisms of SRBD in patients with TMD or the negative effects and underlying mechanisms of TMD in those with SB. Moreover, co-occurrence of SRBDs, such as snoring and OSA and TMD, and other comorbidities, still needs further explanation.

## Conclusions

In this narrative review, the epidemiology, etiology, and pathophysiology of SRBDs, particularly snoring and OSA, including latest research trends, were reviewed. Furthermore, recent research trends and knowledge on the relationship between SRBD and TMD were investigated. In particular, the pathogenesis of OSA involves not only anatomical causes involving the upper airways but also non-anatomical mechanisms such as function of the muscles of the upper airways, arousal threshold, and loop gain. OSA in patients with SRBDs may co-occur with TMD through several mechanisms. In future, studies should examine the specific relationship between SRBDs and TMD.

Typical anatomical features of obstructive sleep apnea (OSA) in orofacial area are: (1) an abnormally enlarged tongue; (2) the long soft palate and/or tonsillar enlargement; (3) an extended uvula; (4) high palatal vault; (5) posteriorly positioned mandible (retrognathia) or small-sized mandible; (6) loss of normal occlusion; (7) adenoid hypertrophy; (8) reduced pharyngeal upper airway space; (9) the increased distance between the mandible and the hyoid bone; and (10) increased neck fat deposition surrounding the upper airway.

## Data Availability

The data supporting the findings of this study are available from the corresponding author, Y-. H.L. upon request.
